# Supplementary material for: IMM-H004 Protects against Cerebral Ischemia Injury and Cardiopulmonary Complications via CKLF1 Mediated Inflammation Pathway in Adult and Aged Rats
Source: Int J Mol Sci. 2019 Apr 3;20(7):1661. doi: 10.3390/ijms20071661 (PMC6480569; doi:10.3390/ijms20071661)
Supplement: Supplementary file 1 [file ijms-20-01661-s001.pdf]

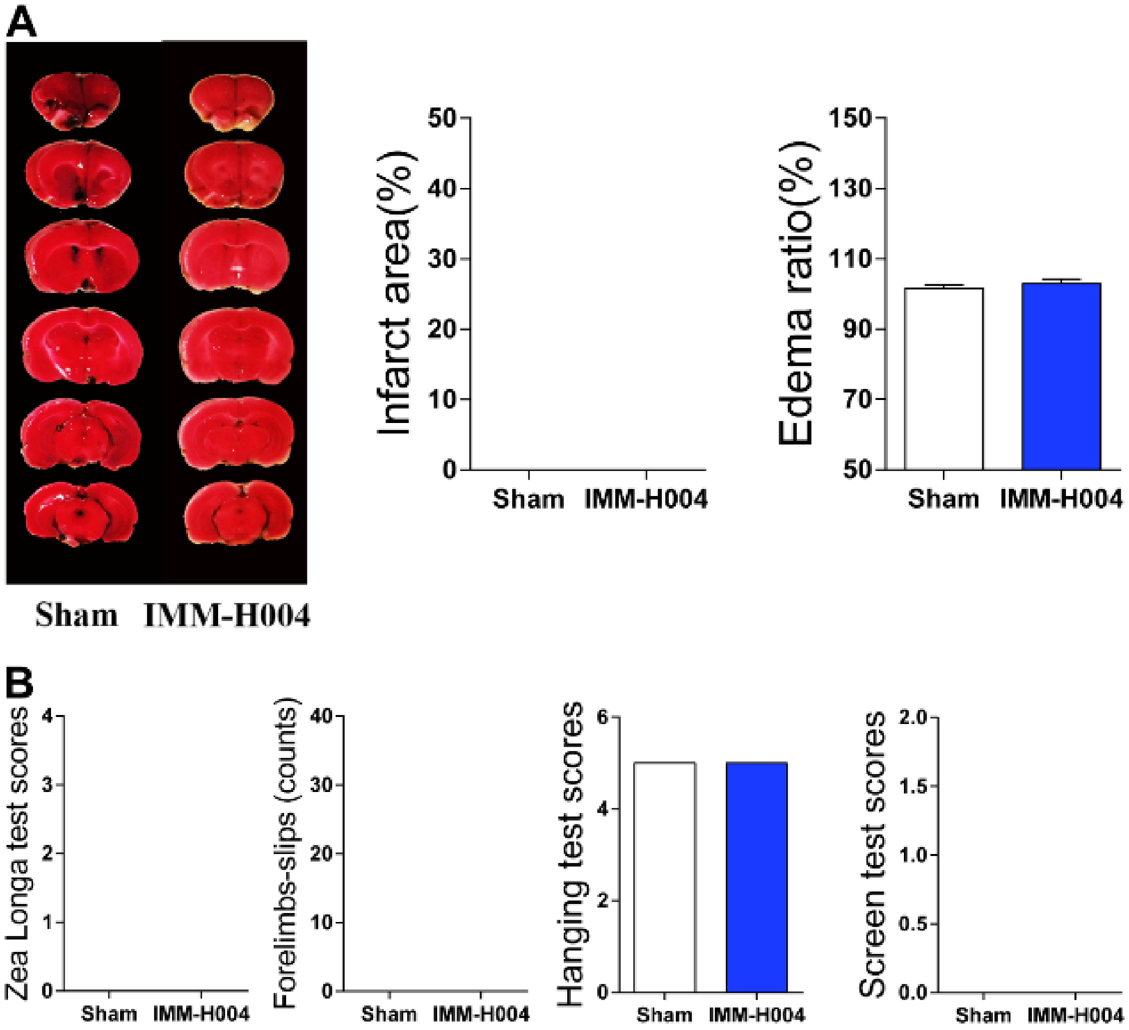

Figure S1. Effect of IMM-H004 on sham group rats. **(A)** Representative images of TTC staining, and analysis of infarction area and edema ratio. **(B)** Statistical analysis of the Zea Longa test scores, Forelimbs-slips, Hanging test scores, and Screen test scores. Data are shown as the mean ± SD (*n* = 10/group).

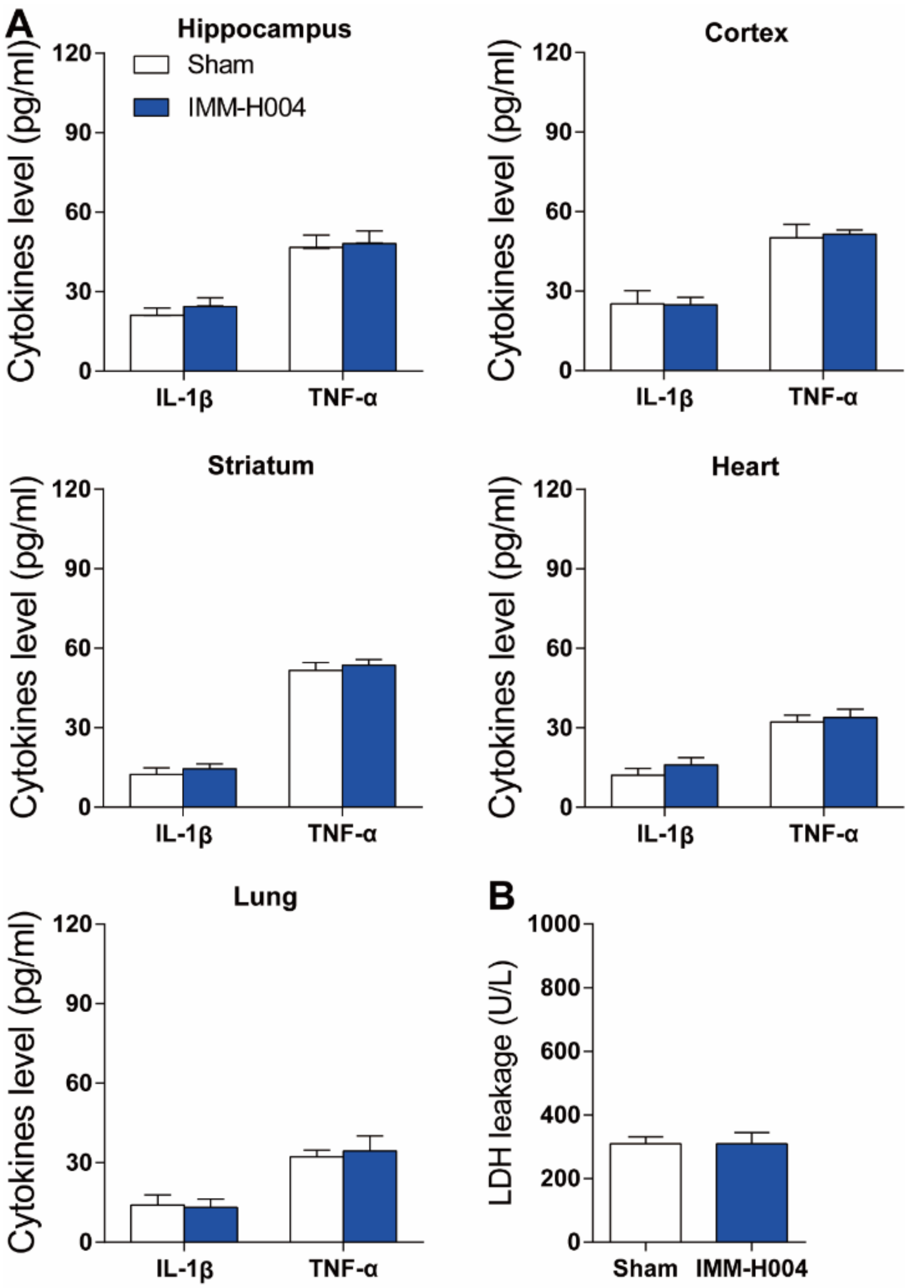

Figure S2. Effect of IMM-H004 on sham group rats. (**A**) Effect of IMM-H004 on IL-1β and TNF-*α* levels in hippocampus, cortex, striatum, heart, and lung of adult rats. (**B**) Effect of IMM-H004 on LDH level in heart of adult rats using an LDH assay kit. Data are shown as the mean ± SD (n = 6/group).
